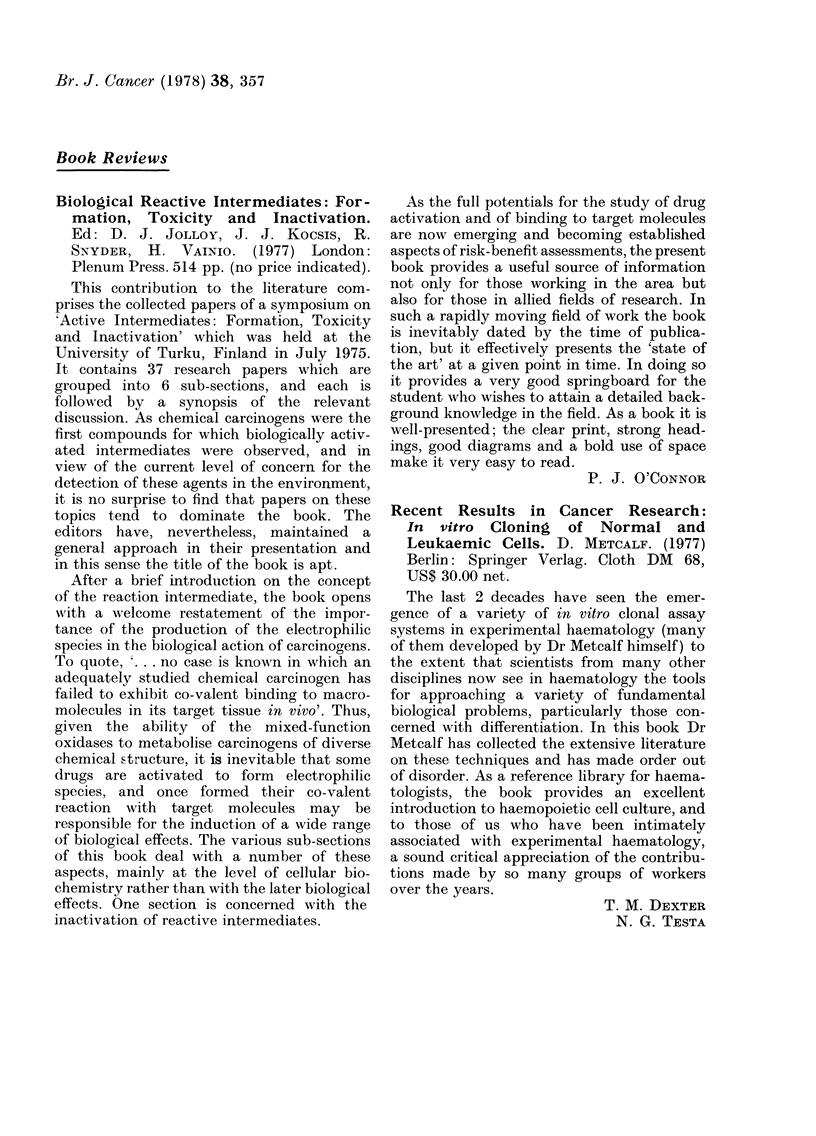# Recent Results in Cancer Research: In vitro Cloning of Normal and Leukaemic Cells

**Published:** 1978-08

**Authors:** T. M. Dexter, N. G. Testa


**Recent Results in Cancer Research: *In vitro* Cloning of Normal and Leukaemic Cells.** D. Metcalf. (1977) Berlin: Springer Verlag. Cloth DM 68, US$ 30.00 net.

The last 2 decades have seen the emergence of a variety of *in vitro* clonal assay systems in experimental haematology (many of them developed by Dr Metcalf himself) to the extent that scientists from many other disciplines now see in haematology the tools for approaching a variety of fundamental biological problems, particularly those concerned with differentiation. In this book Dr Metcalf has collected the extensive literature on these techniques and has made order out of disorder. As a reference library for haematologists, the book provides an excellent introduction to haemopoietic cell culture, and to those of us who have been intimately associated with experimental haematology, a sound critical appreciation of the contributions made by so many groups of workers over the years.

T. M. Dexter
N. G. Testa